# Inflation rate modeling: Adaptive neuro-fuzzy inference system approach and particle swarm optimization algorithm (ANFIS-PSO)

**DOI:** 10.1016/j.mex.2020.101062

**Published:** 2020-09-11

**Authors:** Fateme Nazari Robati, Saeed Iranmanesh

**Affiliations:** Department of Economics, Faculty of Management and Economics, Shahid Bahonar University of Kerman, Kerman, Iran

**Keywords:** Inflation, Unemployment rate, Optimization, ANFIS-PSO

## Abstract

In this paper, modeling was performed using the combination of the ANFIS method and PSO algorithm for the inflation rate in Iran. The data of this article were obtained from the Central Bank of the Islamic Republic of Iran. The raw data are related to the country of the Islamic Republic of Iran and in the period (1986–2018). The purpose of this article is to use the time series data; in the ANFIS system to be trained with the PSO algorithm and using the trained network, a suitable model for production inflation rate be. Inflation is beneficial as an influential variable in economic activity in economic research. Researchers working in macroeconomics, monetary economics, and public sector economics can use the model produced in this paper to analyze inflation formation better.

• We are improving modeling quality by combining ANFIS-PSO.

• Inflation is widely used in economic analysis.

• Inflation rate modeling is a tool for developing anti-inflation programs.

## Graphic abstract

**Figure:**
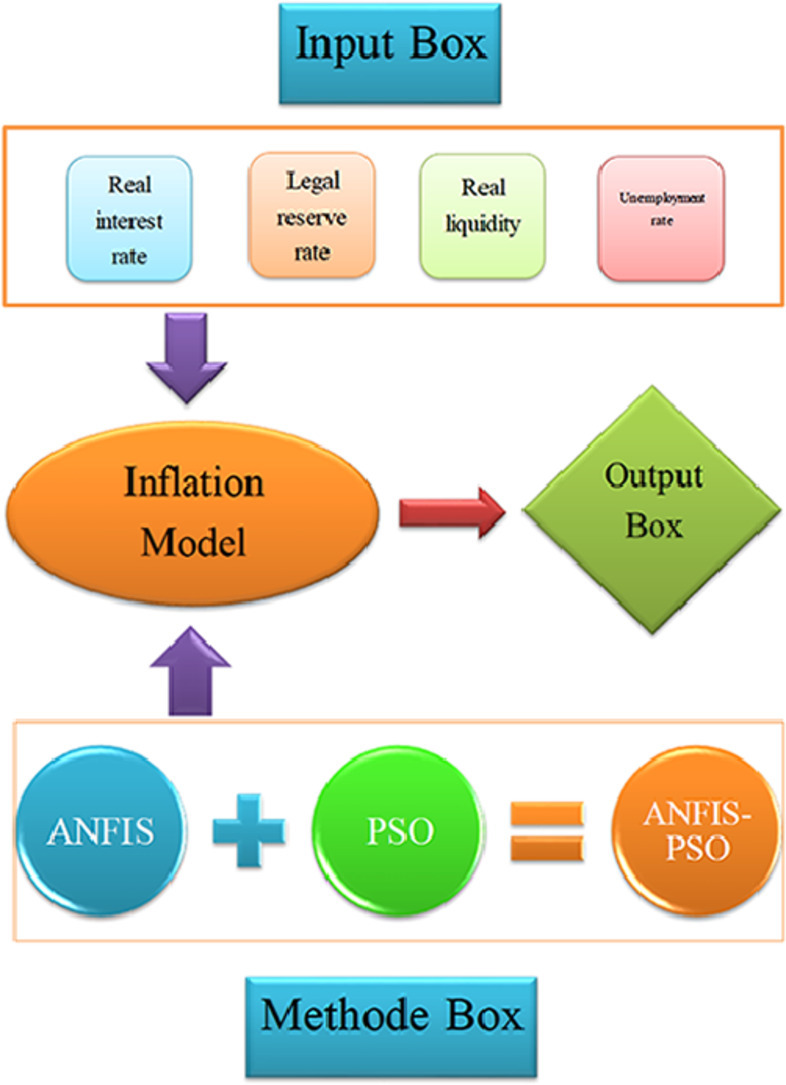

Specifications table

**Table:** 

Subject Area:	Economics and Finance
More specific subject area:	Macroeconomics - Public Sector Economics - Monetary Economics
Method name:	Adaptive Neuro-Fuzzy Inference System With Particle Swarm Optimization Algorithm (ANFIS-PSO)
Name and reference of the original method:	ANFIS: Jang J.S.R., Sun C.T., and M.E. Neuro-fuzzy and Software Computing: a Computational Approach to Learning and Machine Intelligence. In: Prentice-Hall, New Jersey. 1997. PSO Algorithm: Eberhart, R, Kennedy J (1995) A new optimizer using particle swarm theory. In MHS'95 Proceedings of the Sixth International Symposium on Micro Machine and Human Science (pp. 39–43)
Resource availability:	Data source location: http://tsd.cbi.ir Software: MATLAB

## Introduction

One of the significant challenges in the field of economic research today is modeling inflation in countries. Economists are looking at how different variables affect inflation so that they can reduce inflation using appropriate modeling. So far, many classical linear models have been proposed for this subject. But the issue of inflation is a complex and non-linear issue that can not be simulated and analyzed using traditional direct methods. Researchers are always looking for more robust and accurate ways to identify and predict inflation-related parameters by inventing sciences such as smart methods. Artificial neural networks and fuzzy neural networks are suitable tools for estimating and predicting many parameters [1].

In neural networks and fuzzy neural networks, determining the appropriate network structure and selecting their parameters is of particular importance. Also, the success of these networks largely depends on the accuracy and efficiency of their learning algorithms. Various algorithms are used to learn neural networks, the most common of which are gradient-based algorithms, especially post-diffusion and least squares. Although these algorithms have a lot of power, there are some significant weaknesses in them, in some cases create problems for users. Gradient-based algorithms use the local search technique and are always exposed to the optimal local trap. Algorithms such as Levenberg-market (LM) also have high computational complexity [2]. Therefore, the Use of methods to solve problems of gradient-based algorithms has always been of interest to researchers. One of the most appropriate ways is to use collective intelligence algorithms such as particle swarm optimization algorithms for network training. Cooperative intelligence algorithms have a great ability to perform a global search and avoid local optimization [3,4]. In recent years, optimization algorithms have been used to improve the performance of artificial neural network models in modeling and predicting various issues. The ANFIS structure consists of many nodes in different layers that are interconnected [5]. The output of this network depends on adjustable parameters in the nodes. Network learning rules determine how these parameters are updated to minimize errors. The structure of a FIS consists of three main components. Rules database, database, and an argumentation mechanism. A fuzzy rule database contains the IF_THEN rules [6]. The database implements the membership functions used in fuzzy regulations, and the reasoning mechanism, the procedure for inferring the output of input variables. In this paper, the PSO collective intelligence algorithm is used to train the ANFIS network. This attempts to address some of the default algorithms' weaknesses, such as high computational volume and the possibility of being trapped in local optimization. First, the basic structures of the system are done. Then some parameters of PSO algorithm such as number of iterations, number of the initial population, final limit of optimization, coefficients of PSO algorithm, the objective function (in this article MSE), and other parameters related to the algorithm are set. Also, the data is normalized to increase accuracy. The PSO algorithm was then selected as the ANFIS trainer and began training the system. This algorithm tries to find the most suitable model for the inflation rate based on input variables. After modeling, the accuracy of the presented models is checked with statistical indicators.

## Method details

### Adaptive neuro-fuzzy inference system

**Fig. 1:**
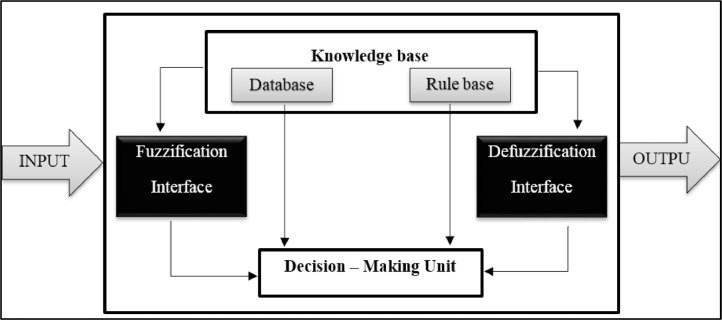
Fuzzy system structure.

Neuro-fuzzy is a hybrid system that combines the ability to make fuzzy logic with the neural network computing and offers a multiple and complex level for modeling. The fuzzy part is about grouping the input data into sets specified by the membership degree, which can be any number between zero and one. The decision for the next activity is based on this set of rules and moving on to the next step. The Adaptive Nervous-Fuzzy inference System (ANFIS) includes parts of a conventional fuzzy system that calculates each step by hidden layers and learning the neural network to increase system information [7]. Jang first introduced ANFIS. This system was used as a practical tool for modeling. This system is similar in performance to fuzzy inference systems [8]. Fig. 1 shows the architecture of an adaptive neural-fuzzy inference system.

The adaptive neural-fuzzy inference system is a hybrid system composed of the ability to make fuzzy logic decisions with neural network computational capability and offers a sophisticated and high level for modeling and Estimation. This system has the advantages of both models. It takes advantage of the trainability of neural networks and the high decision-making power of fuzzy systems in conditions of uncertainty and certainty. The model uses two algorithms of post-error propagation and hybrid method (a combination of descending gradient and error quadratic methods) to train the network, which can reduce the complexity of the algorithm and improve network learning time. Also, the fuzzy inference system used in it is the Sugeno model, which is used to extract fuzzy rules and system output [9].

**Fig. 2:**
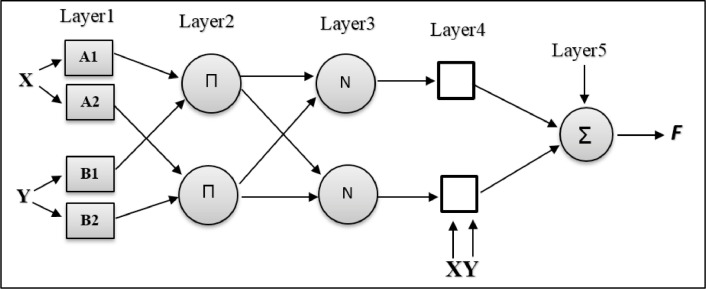
The structure of the Adaptive neural fuzzy inference system.

To further explain the ANFIS model, a fuzzy inference system with two x inputs and one F output is assumed (Fig. 2). For the first time in the Sogno fuzzy model, a rule consisting of a two-phase if-then set of equations (1) is given [10]:

(1) $$\begin{array}{cc} & Rule1:if\;x\;is\;A_{1}\;and\;y\;is\;B_{1}\;Then\;f_{1}=p_{1}x+q_{1}y+r_{1}\\ & Rule2:if\;x\;is\;A_{2}\;and\;y\;is\;B_{2}\;Then\;f_{2}=p_{2}x+q_{2}y+r_{2} \end{array}$$

The node in position *i* of the k-layer is shown as *O*_1,  *i*_ and the node functions in the same layer of the same functional family are as follows: Layer 1 is the input layer, and each node *i* in this layer is a square node with is a membership function (Eq. (2)).

(2) $$\begin{array}{cc} O_{1,i} & =\mu A_{i}\left(x\right)\;for\;i=1,2\\ O_{1,i} & =\mu B_{i-2}\left(y\right)\;for\;i=3,4 \end{array}$$

*O*_1,  *i*_ is a function of the membership of *A_i_*. The input gusset (Eq. (3)) is a maximum of 1 and a minimum of zero. Experimental data and analysis of available data show that this distribution is relatively stable in predicting financial distress. And it makes sense.

(3) $$\mu A_{i}\left(x\right)=e^{-\frac{{\left(x-c\right)}^{2}}{2\sigma^{2}}}$$

In Eq. (3), *C* is the mean, and σ is the variation of the membership function. Each node in layer 2 is a circle node that has a label □. The multiplication of the input signals is expressed by Eq. (4):

(4) $$O_{2,i}=w_{i}=\;\mu A_{i}\left(x\right)\times \mu B_{i}\left(y\right)\;for\;i=1,2$$

Each node in layer three is shown with a circular label. The weights in this path are generally equal to Eq. (5):

(5) $$O_{3,i}={\bar{w}}_{i}=\frac{w_{i}}{w_{1}+w_{2}}$$

Each node *i* in layer 4 enters the membership function corresponding to the same node (Eq. (6)):

(6) $$O_{4,i}={\bar{w}}_{i}f_{i}={\bar{w}}_{i}\left(p_{i}x+q_{i}y+r_{i}\right)\;for\;i=1,2$$

Where *p_i_, q_i_*, and *r_i_* are variable. In this layer, there is a circle node with the Sigma element tag, which is the final output equal to the sum of the inputs (Eq. (7)):

(7) $$O_{5,i}=\sum_{i=1}^{2}{\bar{w}}_{i}f_{i}=\frac{\sum w_{i}f}{w_{i}}$$

Fig. 2 shows a two-input fuzzy inference system that shows the steps of the ANFIS model.

### Decomposition into the main components

Analysis of the main components is a multivariate statistical method. In cases where we have a large amount of information, this method can be used to reduce the complexity of the analysis of variables and better interpret information [11].

By applying this method, the primary variables are converted to new and independent components (with a zero correlation coefficient for both parts). The newly created components are a linear combination of the primary variables. Whit this method, combinations of the initial variable p$,\;x_{1},x_{2},\ldots ,x_{p}\;$ are created to create the independent component *p* (equivalent to the number of primary variables used), i.e.$,\;z_{1},z_{2},\ldots ,z_{p}$.

The lack of correlation between these components reveals different aspects of the primary variables [12].

Each principal component can be identified with a sequence as an Eq. (8):

(8) $$Z_{i}=\alpha_{i1}x_{1}+\alpha_{i2}x_{2}+\ldots +\alpha_{ip}x_{p}$$

Where *Z*_i_ is equal to *i* the main component, *α*_*ij*_ is equal to the coefficients related to the primary variables, *p* is similar to the number of fundamental variables, *x*_*i*_ is equal to the primary variables, and the coefficients related to the primary variables are obtained from solving the Eq. (9):

(9) R−λI=0

Where *I* is equal to the single matrix, *R* is similar to the correlation matrix between the primary variables, and λ equals the specific values. Based on these unique values, particular vectors are obtained.

### Particle swarm optimization algorithm

The particle swarm optimization algorithm is one of the smart optimization techniques based on a social group of birds or fish that randomly search for food in an area. In this algorithm, the idea of which was first proposed by Eberhart and Kennedy (1995), hypothetical living organisms (birds or fish) are called Particle [13].

Each of these Particles has five properties. The position is the objective function corresponding to this position, speed, best position, and the amount of objective function corresponding to the best position experienced. in the algorithm, the location and speed of each Particle are according to Eqs. (10) and (11) . and based on the information of the previous step.

(10) $$X_{i}^{j}\left[t+1\right]=X_{i}^{j}\left[t\right]+V_{i}^{j}\left[t+1\right]$$

(11) $$V_{i}^{j}\left[t+1\right]=WV_{i}^{j}\left[t\right]+C_{1}r_{1}\left(X_{p,best}^{j}\left[t\right]-X_{i}^{j}\left[t\right]\right)+C_{2}r_{2}(X_{g,best}\left[t\right]-X_{i}^{j}\left[t\right]$$

**Fig. 3:**
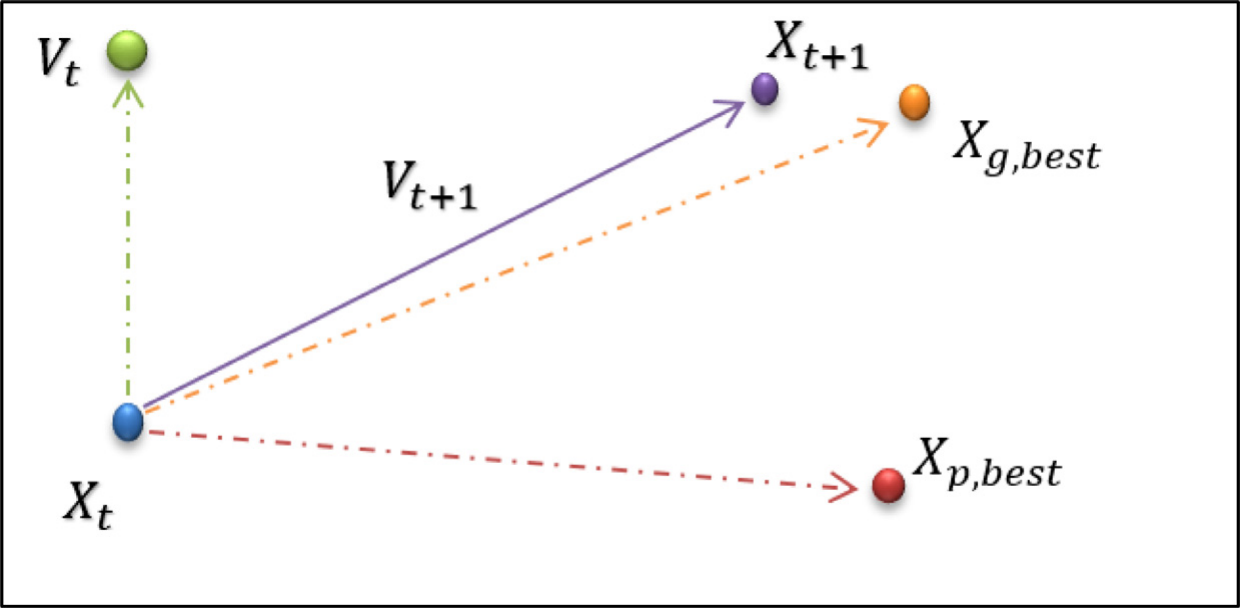
Particle orientation in the PSO algorithm.

W is the inertia coefficient, *r*_1_ and *r*_2_ are random vectors with uniform distribution in the range (1 and 0), *C*_1_ and *C*_2_ are the Particle learning coefficient and the collective learning coefficient in the range (2 and 0), $X_{p,best}^{j}$, respectively. Particle and *X*_*g*,  *best*_ are the best-combined situations experienced? Thus, the new Particle position is a combination of moving in the direction of the previous velocity, the best experienced personal area of the Particle, and the best-experienced position of the sum, which can be seen in Fig. 3.

This law of motion is fixed for all Particles. Finally, during these collaborations and according to the defined criteria for stopping, all Particles reach the optimal answer to the desired problem. The PSO algorithm was used to improve the performance of the ANFIS system. First, the particle vectors' initial values and velocity vectors must be determined using a completely random distribution function. It should be noted that random values are first represented by a computer and are defined in the range of zero and one. In the first step, each Particle is generated by multiplying a random number in the parameter. The production of random values of the settings in the form mentioned above is significant because the range of values of the model parameters is entirely different from each other. Choosing a series of random parameters that are not within the acceptable range can stop the model fail. Finding leads to convergence. Therefore, it can be said that although the initial values of the Particles are randomly selected, this selection must be such that the parameters are within acceptable limits. The benefits of the settings made in the first iteration are introduced separately to the model. This introduction is done by writing the values mentioned in one of the input files of the model, which includes its parameters.

It should be noted that at each time step, *n* Particle numbers are checked. Therefore, the ANFIS, *n* bar model must be executed at any time step. Each time the model is implemented, a time series of inflation is simulated and introduced as output. The calculated values for simulation inflation are compared with the observed inflation, which is constant. Thus, after the first time step, the *n* bar model is executed. So that in the first step, one value is obtained for each Particle for the objective function. In the first iteration, the pbest for each Particle is the same as the current Particle state. The gbest, which is the best Particle encountered position in the swarm set, is determined by comparing the objective values of each Particle and selecting the maximum amount. In the second iteration, using pbest and gbest values and the status and velocity values of the previous stage particles (first repetition) and based on Eq. (12), the new stage's speed is calculated for the Particle. Then, according to Eq. (13), the latest state of the Particles is determined.

(12) $$V_{id}^{n+1}=\left[WV_{id}^{n}+C_{1}r_{1}^{n}\left(p_{id}^{n}-X_{id}^{n}\right)+C_{2}r_{2}^{n}\left(p_{gd}^{n}-X_{id}^{n}\right)\right]$$

(13) $$X_{id}^{n+1}=X_{id}^{n}+V_{id}^{n+1}$$

**Fig. 4:**
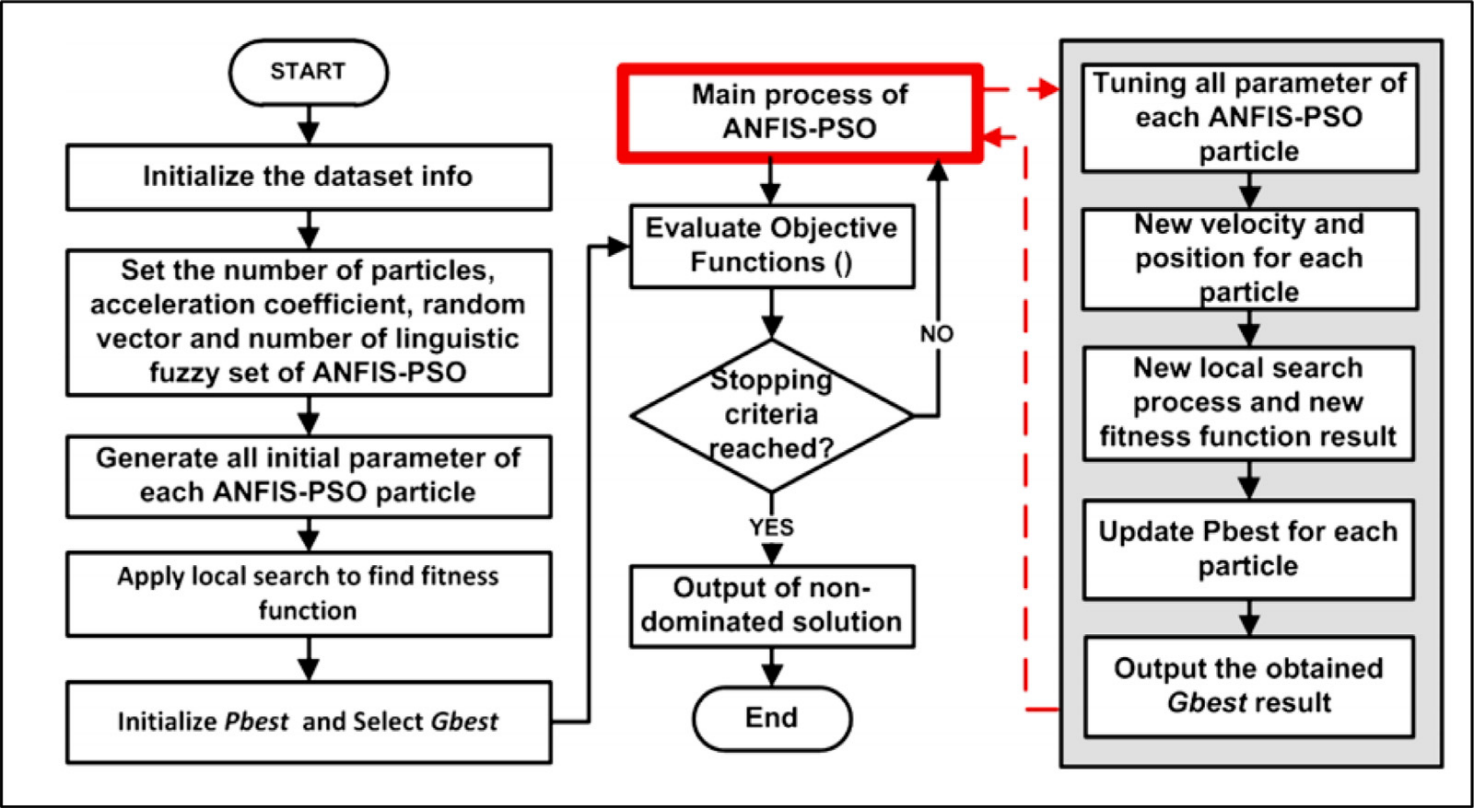
Flowchart ANFIS-PSO Method.

Then, in the second iteration, just like the first iteration, the values of the objective function for each Particle are created. By comparing these values with the values obtained in the first iteration, the pbest is determined for each Particle. If the situation encountered by Particle in the new stage were better than the situation faced in the previous step, the pbest for that Particle would also be the best situation. And this situation is considered as the best case met for all Particles as gbest at this stage. In this way, the gbest and pbest in each step are made from the values of the previous step, and the values of the velocity and velocity vectors in each step are updated according to the position of the Particle in the last step. Finally, at the end of the previous iteration step, the previous gbest step is introduced as the best position and the corresponding inflation value as the answer. The ANFIS-PSO flowchart is as shown in Fig. 4 [14].

Factors affecting the inflation rate are expressed according to Eq. (14).

(14) y=f(x1,x2,x3,x4)

**Fig. 5:**
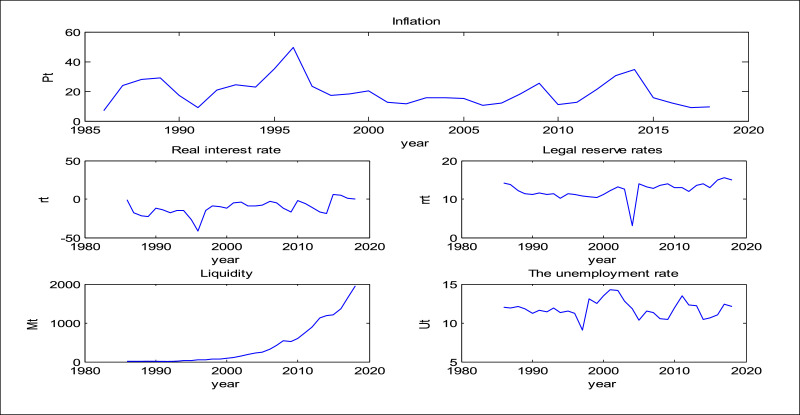
Charts for model variables.

Y represents the annual inflation rate, and the values x1 to x4 represent the real interest rate, legal reserve rate, real liquidity, and the unemployment rate over the period (1986–2018). The costs for the trend of these variables are shown in Fig. 5. Modeling of the inflation rate citizenship form in Iran was performed using the ANFIS-PSO method by MATLAB software.

### Criteria for evaluating results

The accuracy of the model obtained in this study was investigated based on statistical indicators indicating the Error. These indicators are introduced according to the equations of 15–20.

(15) $$Mean\;Square\;Error\;MSE=\frac{1}{33}\sum_{i=1}^{33}{\left(Target_{i}-output_{i}\right)}^{2}\;$$

(16) $$Root\;Mean\;Square\;Error\;RMSE=\sqrt{\frac{1}{33}\sum_{i=1}^{33}{\left(Target_{i}-output_{i}\right)}^{2}\;}\;$$

(17) $$Mean\;Absolute\;Error\;MAE=\sqrt{\frac{1}{33}\sum_{i=1}^{33}\left|Target_{i}-output_{i}\right|\;}\;$$

(19) $$Coefficient\;of\;Determination\;R^{2}={\left(\frac{\sum \left(Target_{i}-\bar{Target}\right)\;\times \;\left(output_{i}-\bar{output}\right)}{\sqrt{\sum {\left(Target_{i}-\bar{Target}\right)}^{2}\times \sum {\left(output_{i}-\bar{output}\right)}^{2}}}\right)}^{2}$$

(20) $$Error\;standard\;deviation\;STD\;Error=\sqrt{\frac{1}{33}\sum_{i=1}^{33}(Target_{i}-outpu{t_{i)}}^{2}}$$

## Results

For the present study, the data were divided into two groups: Test Data and Train Data. 60% of the data is in the Train Data group, and the rest is in the Test Data group. Data were randomly selected for grouping. The target function was considered to be the difference between Target values as real values and Output values as calculated values. Eq. (21) shows the objective function.

(21) $$Z=\sum_{i=1}^{33}Target_{i}-output_{i}$$

**Fig. 6:**
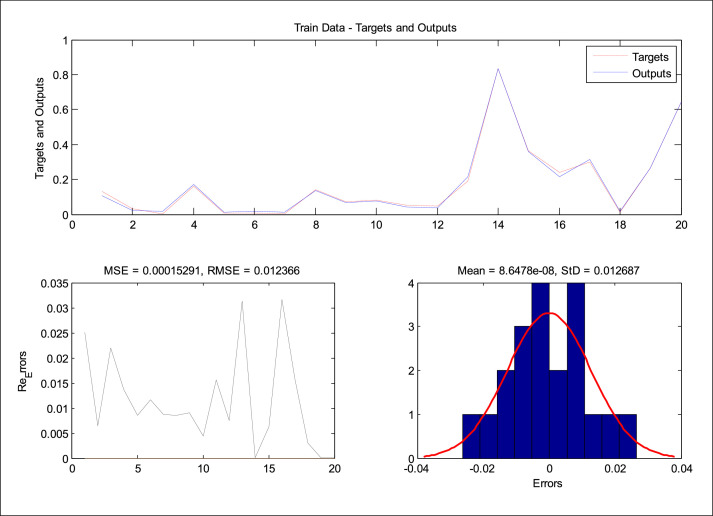
Modeling results on training data.

After running ANFIS-PSO, the results obtained for Train Data values are by Fig. 6. Target and output values were plotted. In this figure, the performance of the system can be observed. Also, the error values for Train Data are calculated in this figure.

**Fig. 7:**
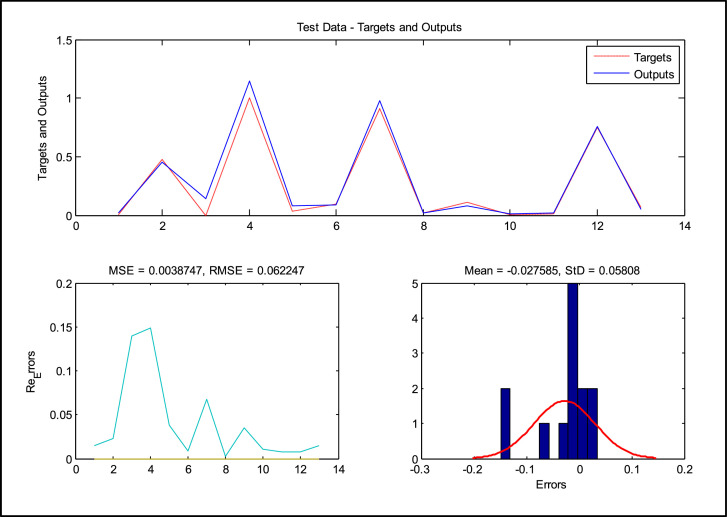
Modeling results on testing data.

For the second group of data in the form of Test Data output, the ANFIS-PSO method is by Fig. 7. In this figure, the observed and actual process of the data and the model measurement criteria are given.

**Fig. 8:**
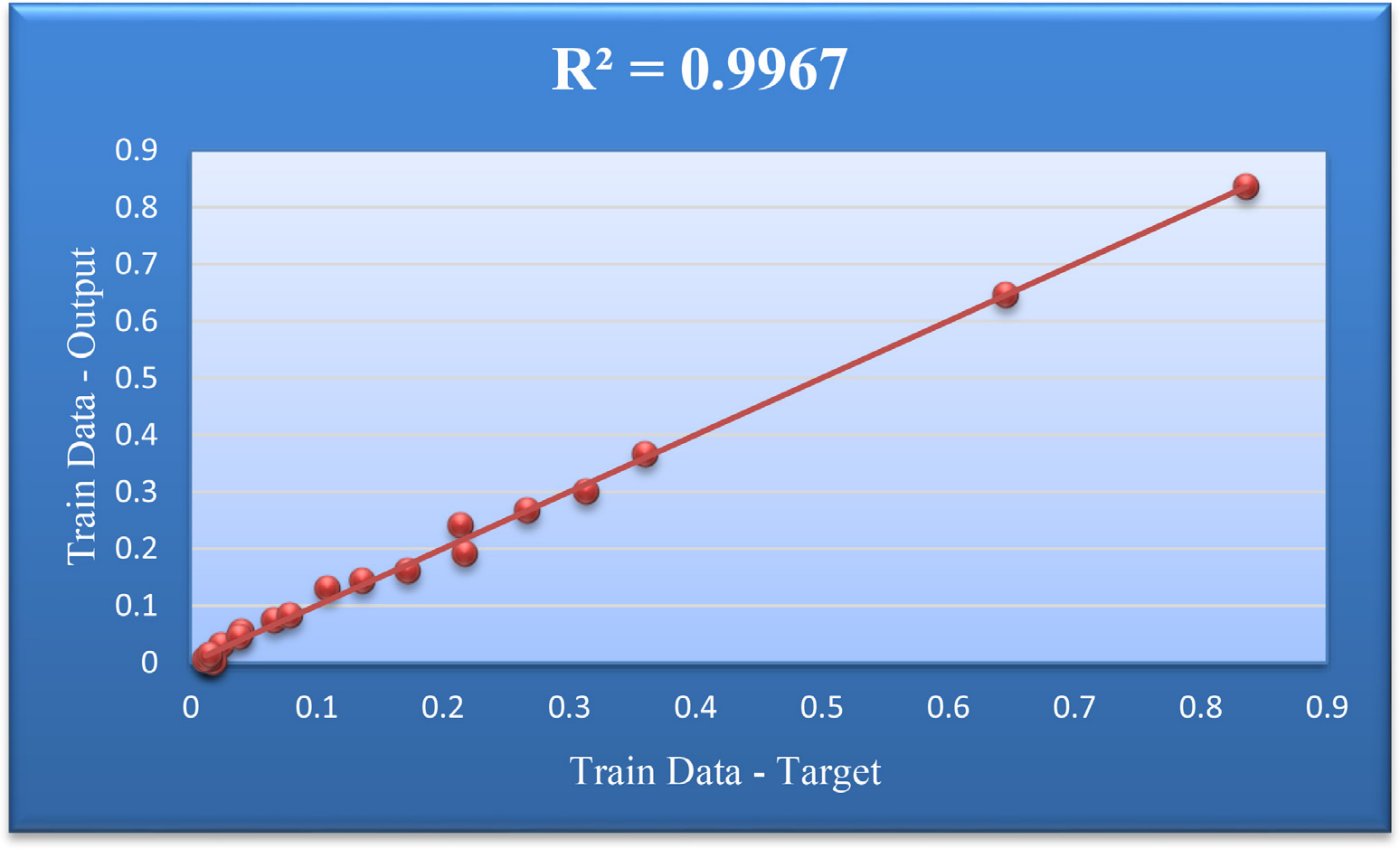
Regression target and output values for testing data.

Fig. 8 was drawn to obtain the Coefficient of Determination for the Train Data group. In this figure, the values of Target and Output for Train Data are plotted in one form, and the amount of R^2^ is reported.

**Fig. 9:**
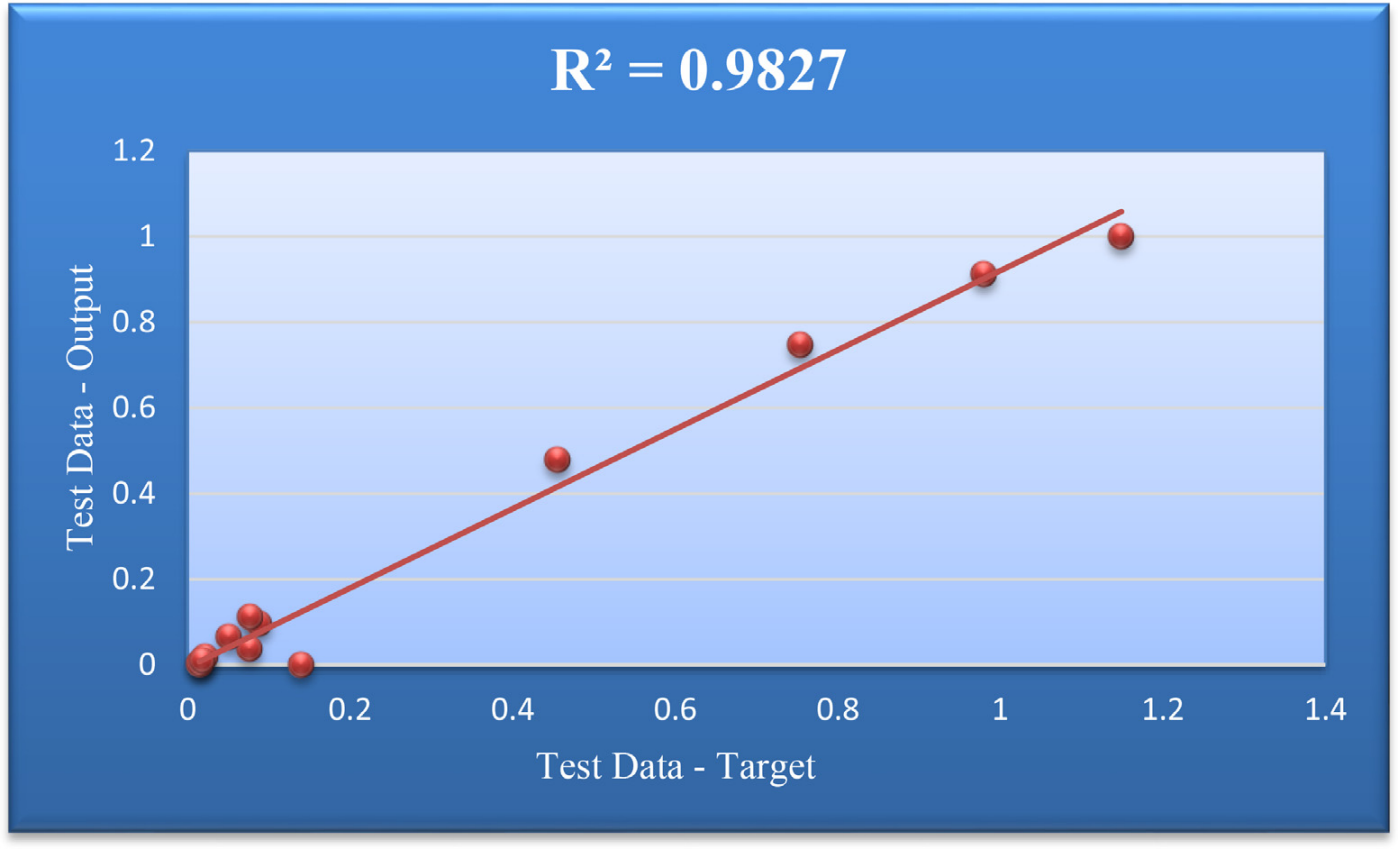
Regression target and output values for training data.

Also, Fig. 9 was drawn to obtain the Coefficient of Determination for the Test Data group. In this figure,for test data, output values, target, and statistics R2 are specified.

**Fig. 10:**
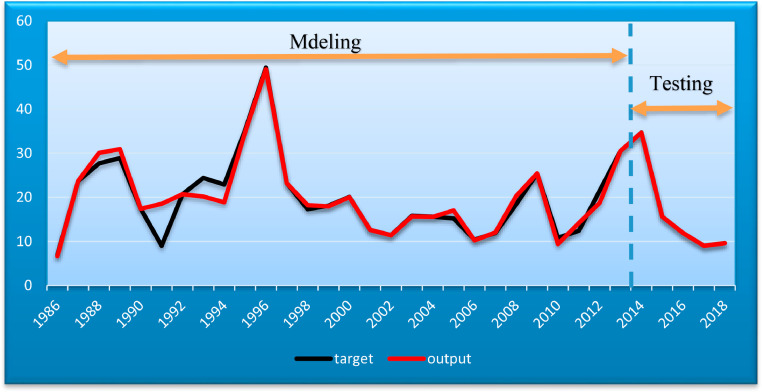
Comparison of target values and modeling output.

In the next section, the total data of the model was evaluated using the constructed network. Fig. 10 shows the Target and Output values of the ANFIS-PSO system for the inflation model. The degree of conformity of the values obtained from the modeling and the actual costs are shown in this figure. The final five years of data were used to evaluate the built model.

Table 1 shows the relative error values in the final five years of model evaluation.

**Table 1:** Relative error values of model evaluation data.

Model testing	2014	2015	2016	2017	2018	Average
Target Data	34.7	15.60	11.90	9.00	9.60	–
Output Data	34.65	15.63	11.89	9.01	9.58	–
Relative Error (%)	0.13	0.20	0.03	0.13	0.10	0.12

**Fig. 11:**
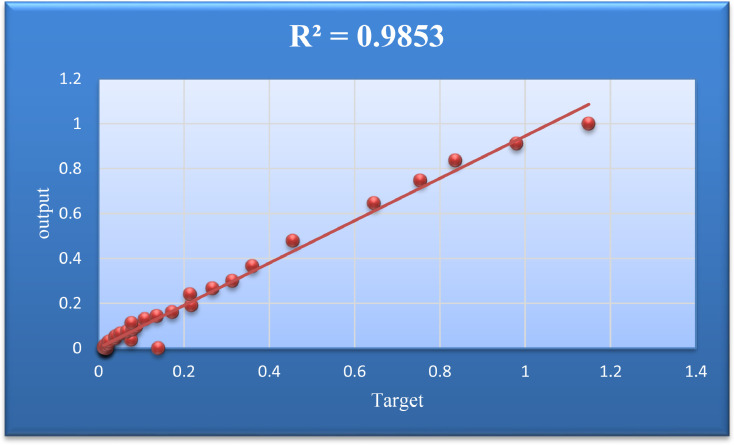
Target and output data regression.

Fig. 11 shows the Coefficient of Determination values for the whole model.

Table 2 shows the values of the model evaluation indicators in this study.

**Table 2:** Performance appraisal of the ANFIS-PSO model for inflation.

*Model evaluation criteria*		Total Data	Train Data	Test Data
*Mean Square Error*	*MSE*	0.0016	0.0001	0.0038
*Root Mean Square Error*	*RMSE*	0.0402	0.0123	0.0622
*Mean Absolute Error*	*MAE*	0.0215	0.0095	0.0400
*Coefficient of Determination*	**R**^2^	0.9853	0.9967	0.9827
Error standard deviation	STD	0.0344	0.0126	0.0580

## Declaration of Competing Interest

The authors declare that they have no known competing financial interests or personal relationships that could have appeared to influence the work reported in this paper.

## References

[1] Moayedi H., Raftari M., Sharifi A., Jusoh W.A.W., Rashid A.S.A. (2020). Optimization of ANFIS with GA and PSO estimating α ratio in driven piles. Eng. Comput..

[2] Alsarraf J., Moayedi H., Rashid A.S.A., Muazu M.A., Shahsavar A. (2020). Application of PSO–ANN modelling for predicting the exergetic performance of a building integrated photovoltaic/thermal system. Eng. Comput..

[3] Turen U., Yunus G., Ahmet K. (2016). National ICT, economic freedom and human development. Acad. J. Article.

[4] Georgiou M.N. (2015). Economic freedom and human development index. Econ. Res. Netw..

[5] Petković D., Nikolić V., Mitić V.V., Kocić L. (2017). Estimation of fractal representation of wind speed fluctuation by artificial neural network with different training algorothms. Flow Meas. Instrum..

[6] Sedghi Y., Zandi Y., Shariati M., Ahmadi E., Moghimi Azar V., Toghroli A., Safa M., Tonnizam Mohamad E., Khorami M., Wakil K. (2018). "Application of ANFIS technique on performance of C and L shaped angle shear connectors. Smart Struct. Syst..

[7] Citakoglu H. (2014). Estimation of monthly mean reference evapotranspiration in Turkey. Water Resour. Manag..

[8] Jang J.-S.R., Sun C.-.T., Mizutani E. (1997). Neuro-fuzzy, and soft computing, a computational approach to learning and machine intelligence [Book Review]. IEEE Trans. Autom. Control.

[9] Singh R., Kainthola A., Singh T. (2012). Estimation of elastic constant of rocks using an ANFIS approach. Appl. Soft Comput..

[10] Zadeh L.A. (1965). Fuzzy sets. Inf. Control.

[11] Çamdevýren H. (2005). Use of principal component scores in multiple linear regression models for prediction of Chlorophyll-a in reservoirs. Ecol. Modell..

[12] Morrison, D.F., L.C. Marshall, and H.L. Sahlin, Multivariate statistical methods. 1976.

[13] Eberhart R., Kennedy J. (1995). A new optimizer using particle swarm theory. in MHS'95. Proceedings of the Sixth International Symposium on Micro Machine and Human Science.

[14] Rini D.P., Shamsuddin S.M., Yuhaniz S.S. (2016). Particle swarm optimization for ANFIS interpretability and accuracy. Soft Comput..

